# Hypertrophic synovial plica in the lateral side of the knee - A case report: Case report and literature review

**DOI:** 10.1016/j.ijscr.2024.109705

**Published:** 2024-04-25

**Authors:** Arash Sherafatvaziri, Fardis Vosoughi, Alireza Mirzamohamadi, Erfan Babaei Nejad, Ramin Shayan-Moghadam

**Affiliations:** a Department of Orthopedic Surgery, Shariati Hospital, School of Medicine, Tehran University of Medical Sciences, Tehran, Iran; b Department of Orthopedic and Trauma Surgery, Center for Orthopedics Trans-Disciplinary Applied Research, Shariati Hospital, Tehran University of Medical Sciences, Tehran, Iran; c Department of Orthopedic Surgery, Shariati Hospital, Tehran University of Medical Sciences, Tehran, Iran; d Department of Orthopedic Surgery, Center for Orthopedic Trans-disciplinary Applied Research, Shariati Hospital, Tehran University of Medical Sciences, Tehran, Iran

**Keywords:** Arthroscopy, Knee, Knee joint, Lateral Plica, Plica, Synovial Plica

## Abstract

**Introduction:**

Plicae or synovial folds can be detected in different joints, especially around the knee. Synovial plicae pathologies are rare conditions with difficulty in diagnosis because of various symptoms overlapping with other diseases.

**Presentation of case:**

We reported a rare case of symptomatic hypertrophic synovial plica in the lateral side of the knee in a 12-year-old boy following a traumatic event almost two years before the surgery. The diagnosis and treatment were conducted by knee arthroscopy, and follow-up of the patients showed significant improvements with no pain or range of motion restrictions.

**Discussion:**

The reported case had a significantly lower age of presentations compared to most previously reported cases, and he was diagnosed with lateral knee hypertrophic plicae, while medial knee hypertrophic plicae are more commonly reported, which is considered rare findings. Contrary to previous studies of lateral plica, our case had a history of significant direct trauma, and he was not a professional athlete. Furthermore, based on evidence, hypertrophic synovial plicae are mostly asymptomatic, but in our case, there was a pain in his knee that worsened in flexion.

**Conclusion:**

Physicians should consider the possibility of synovial hypertrophic plicae, especially in younger patients with histories of direct traumatic events.

## Highlights

• knee hypertrophic plicae are rare and often asymptomatic conditions that are found accidentally during knee arthroscopic evaluations.
• Based on evidence, hypertrophic synovial plicae are usually asymptomatic but our case experienced localized pain and tenderness in the knee with increased severity in flexion.
• This case presented at a younger age than previous cases and was diagnosed with lateral knee hypertrophic plicae, which is less commonly reported than medial knee hypertrophic plicae.
• Contrary to previous studies of lateral plica, our case had a significant direct trauma and he was not a professional athlete.
• Physicians should consider the possibility of synovial hypertrophic plicae, especially in younger patients with histories of direct traumatic events.

## 1 Introduction

Plicae or synovial folds could be detected in different joints, especially around the knee. It is a synovial membrane that lies between the suprapatellar pouch and the knee [1,2]. It can be present with anterior knee pain, clunking, clicking, and a popping sensation during activities involving the patellofemoral joint, such as squatting. Depending on their anatomical location, there are four types of synovial plicae: suprapatellar, mediopatellar (which is the most commonly symptomatic one), infrapatellar, and lateral [3]. The exact mechanism of synovial plicae development is still unclear, but it has been suggested that the etiology of this condition lies in defects during embryogenesis [4].

Synovial plicae pathologies are rare conditions with difficulty in diagnosis because of various symptoms overlapping with other diseases.

There are few available data on the prevalence of hypertrophic plicae because it is very rare, and its diagnosis requires special attention [5].

The most commonly reported presentation of pathologic synovial plicae is regional pain exacerbated by activity, particularly crouching or kneeling. The main goal of treating plica syndrome is to slow or prevent the destructive process. The therapeutic management of hypertrophic knee plicae relies on conservative and surgical techniques [6]. Conservative therapy offers various medications but may not work for inflammatory reactions in plica syndrome. Conservative treatment often fails, with success rates as low as 0 %–16 %, particularly in older patients. Surgical removal of synovial plica is preferred in such cases. Arthroscopic resection effectively reduces pain, enhances mobility, and alleviates signs of synovitis [7]. In the current study, which is reported in line with the SCARE criteria [8], we present a 12-year-old boy with a diagnosis of hypertrophic knee plicae and treated by arthroscopic method. Written informed consent was obtained from the patient and his parents to publish this case report and accompanying images. Furthermore, we conducted a systematic literature review of lateral plica and summarized similar synovial lateral plica cases.

## 2 Case presentation

The present case is a 12-year-old boy who was admitted to the orthopedic department of our medical center due to chronic and severe pain in his left knee. He was not a professional athlete, and his activity level was typical for an adolescent. The parents stated a blunt traumatic injury to the affected knee about two years ago that triggered the knee pain in the patient. He had undergone conservative treatments, including non-steroidal anti-inflammatory drugs (NSAIDs) for the past six months and also a single dose of lidocaine and corticosteroid intra-articular injection three months ago that did not result in significant persistent improvements but temporarily relieved the pain.

During the physical examination of the lower limbs at the time of admission, normal skin was observed without signs of injuries. There was a regional pain in the supra-lateral site of the patella in the 90° flexion position during ROM examination that was relieved with more flexion or extension. Moreover, point tenderness was felt in the lateral side of the patella directly on the condyle in the knee 90° flexion position. The patient's pain was not related to the activities. During the knee range of motion (ROM) examination, a mild click was also found when going from full extension to flexion at 100°. Knee ROM was 0–130°.

**Fig. 1:**
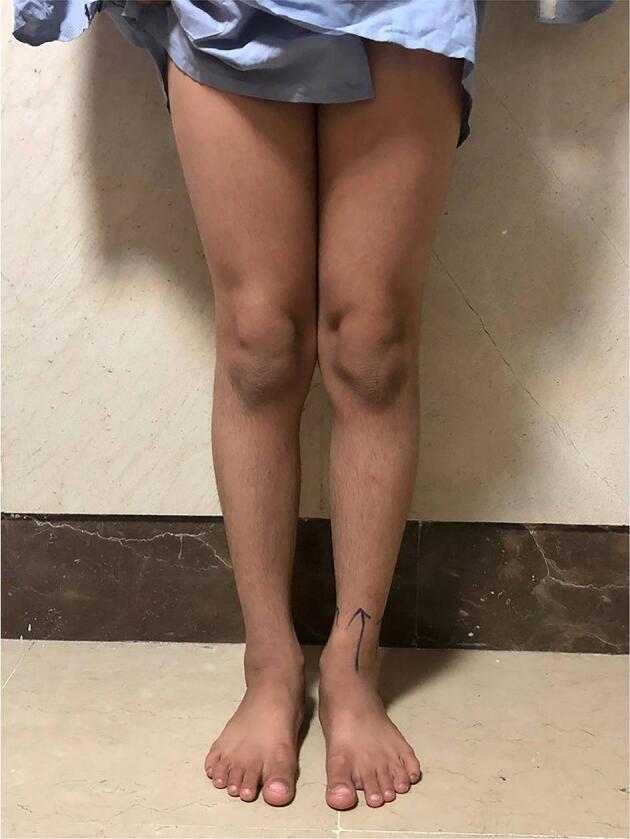
Patient's both knee clinical appearance.

The hyperflexion and hyperextension of the knee were associated with no pain. Knee stability was preserved in the valgus stress test and varus stress test. The extensor mechanism was normal, no mal-alignment was observed, and the patient's gait was intact. Other examinations, including pivot, McMurray, and Lachman tests, were normal. Neuromuscular examinations of lower limbs were normal. The patient's knee clinical appearance is shown in Fig. 1.

**Fig. 2:**
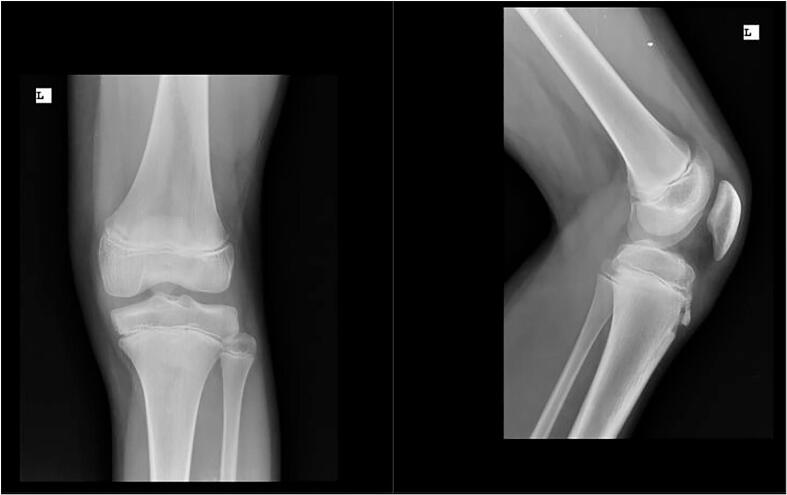
The X-ray of the affected knee.

**Fig. 3:**
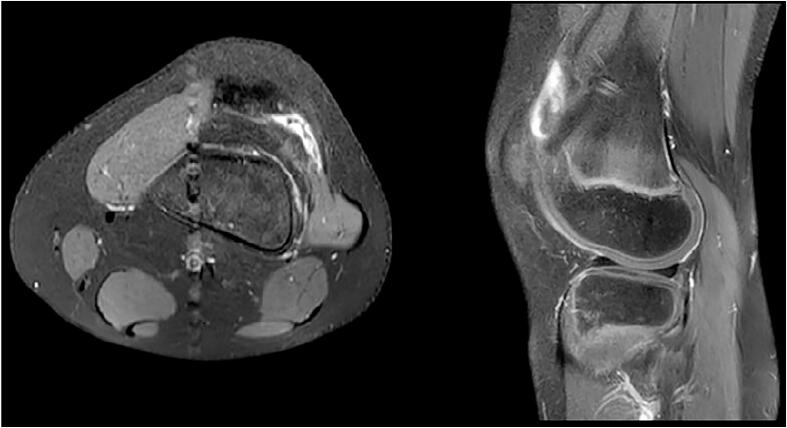
MRI of the left knee showing significant hypertrophy of the synovial membrane (a thin hypointense band lateral to the lateral femoral condyle).

The X-ray of the affected knee indicated no pathologies (Fig. 2), but the left knee MRI showed significant hypertrophy of the synovial membrane (Fig. 3).

The patient underwent knee arthroscopy with suspicion of hypertrophic plica. A hypertrophic synovial plica in the lateral side of the left knee was observed. A complete resection of hypertrophic plica was performed. The surgery details are as follows: A systematic examination of the knee joint was performed. The patellofemoral, medial, and lateral compartments were evaluated for any signs of pathology. Cartilage surfaces, menisci, ligaments, and synovium were inspected thoroughly. Attention was directed towards the lateral compartment, specifically the lateral plica. The lateral plica was identified and assessed for any abnormalities, including thickening, fraying, or impingement. Subsequently, arthroscopic shavers and scissors were used carefully to resect the affected portion of the plica. Great care was taken to preserve surrounding structures and minimize trauma to the synovium. After resection, the knee joint was thoroughly irrigated with saline solution to remove debris. The arthroscope was used to confirm the successful resection of the lateral plica and to ensure the absence of any residual pathology.

**Fig. 4:**
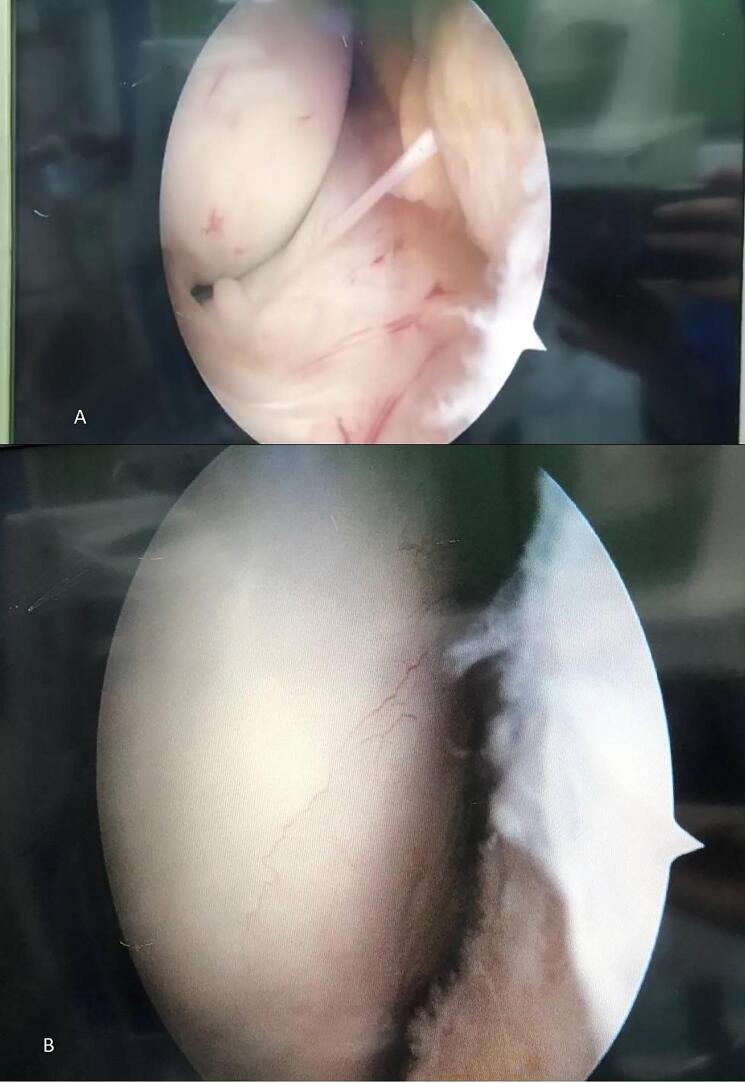
Arthroscopy findings indicating hypertrophic synovial plica in the lateral side of the left knee. A: hypertrophic synovial plica. B: angiogenesis of the plica in the femoral condyle.

Moreover, an accidental finding of the knee arthroscopy was angiogenesis in the femoral condyle beneath the hypertrophic plica (Fig. 4). Angiogenesis is commonly found in pathologic plica, caused by inflammation due to friction, with increased inflammatory cells and hyalinization [9].

**Fig. 5:**
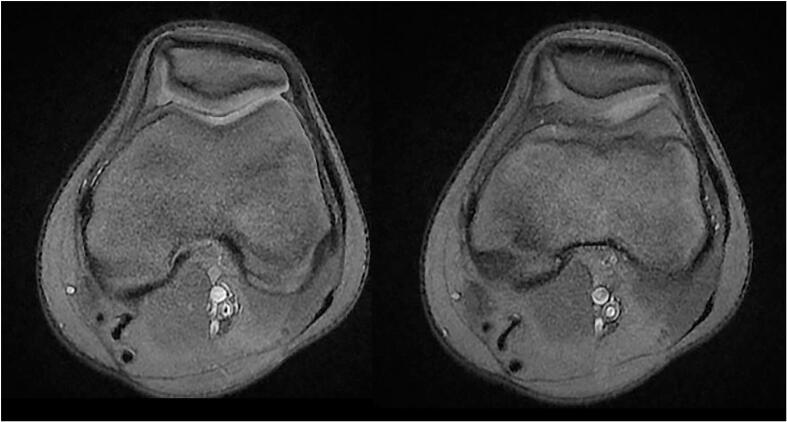
MRI of the left knee showing no plica (1.5 years after arthroscopic resection).

The patient's follow-up immediately and after six months showed complete remission without further knee pain. No ROM restrictions or complications are reported during flexion from 0° to 140°. Moreover, in the recent follow-up visit of this patient, 1.5 years after surgical resection, he did not suffer from any pain in the rest and physical activity. He also had a full knee range of motion without any limitation or lag. Fig. 5 shows the follow-up MRI.

## 3 Discussion

As explained earlier, knee hypertrophic plicae are rare and often asymptomatic, typically found accidentally during knee arthroscopic evaluations. To date, only limited studies have been conducted on the prevalence and clinical impacts of knee hypertrophic plicae. In this study, we present a 12-year-old boy with presentations of localized pain and tenderness in the knee with increased severity in flexion who was diagnosed with lateral knee hypertrophic plicae and treated by arthroscopic resection.

In 2014, Schindler and colleagues reviewed and analyzed the data of previous reports on synovial plica and outcomes of open or arthroscopic plica excisions. By evaluating 969 patients, they showed that trauma was the most common cause of symptomatic plica development in 57 % of cases. The improvement rate of plica excision was reported to be 90 % [9]. Another study was conducted by Gurbuz and colleagues in 2006. In this survey, they evaluated 318 knee arthroscopies through video records. They reported that lateral synovial plicae are less common than other sites, and most cases are adults over 20 years old [10]. Most reported hypertrophic synovial plicae cases are on the medial side. However, Kurosaka and colleagues reported the hypertrophic synovial plicae in the lateral knee in a 21-year-old male. In this case, the patient reported significant pain in the affected knee but no history of traumatic accidents [11]. In another study by Kanazawa and others in 2017, they reported bilateral symptomatic lateral parapatellar synovial plica of the knee. The reported case was a 15-year-old competitive soccer player who underwent diagnostic and therapeutic actions via arthroscopy [12]. These data highlight the importance of our case presentations. As we described, our case had a previous history of direct traumatic injury to the affected knee, and symptoms appeared during the next two years. Studies suggest that Plicae are residual structures from the embryological formation of the knee. These structures can undergo inflammation, thickening, and symptomatic manifestation after blunt trauma and irritation [13,14]. We hypothesize that the patient had a congenital knee plica. Subsequently, the blunt trauma initiated the inflammatory response and thickening of the plica, resulting in pain that persisted for two years. As a significant notion compared to other reported cases, this case had a lower age of onset. Besides, he was diagnosed with lateral knee hypertrophic plicae which is less commonly reported in the literature compared to medial knee hypertrophic plicae. Therefore, we believe that this reported case could have high clinical value.

Blok and colleagues also assessed the epidemiology and characteristics of knee plicae. After reviewing data from 912 arthroscopies, they showed that medial knee synovial plicae could be observed in up to 21.8 % of cases in some centers. They also stated that plica syndrome is a significant clinical problem involving young people, especially women [15].

In this study, we observed a hypertrophic synovial plica in the lateral side of the knee in a 12-year-old boy. Both the position of the synovial plica and the patient's age could be considered rare findings. Furthermore, based on evidence, hypertrophic synovial plicae are mostly asymptomatic [[16], [17], [18]], but our case had pain in his knee that worsened in flexion. These data are considered rare findings of this case. One factor aiding diagnosis was the patient receiving a single dose of lidocaine and corticosteroid injection three months before surgery, which temporarily relieved pain and indicated the pain's intra-articular nature.

Treatments for this disease usually involve either intra-plical or intra-articular corticosteroid injections or arthroscopic surgical excisions. Up to this point, no innovative aspects or novel techniques have been utilized in the arthroscopic intervention to manage plica in the literature. Another treatment approach is structured physiotherapy, but arthroscopic resection has a better outcome. High compliance to self-conducted training is crucial for long-term success in structured physiotherapy; therefore, surgery is a better choice if it fails or compliance is lacking [13].

Our case was treated by arthroscopic resection after intra-articular corticosteroid treatment failed. Arthroscopic resection of hypertrophic plicae is considered safe and highly effective, especially in the early stages. Former studies have reported better outcomes in young patients with localized symptoms and early management.

To provide a comprehensive overview, we systematically searched electronic databases, including PubMed (MEDLINE), Scopus, Web of Science, and Google Scholar, up to November 27, 2022. The following search strategy was used:

("Lateral plica" OR "Lateral Para patellar plica" OR "Plica” OR "Synovial plica" OR "Lateral plica syndrome" OR "Plica syndrome" OR "Plica, synovial" OR "Synovial plicae" OR "Synovial fold" OR "Fold, synovial" OR "Synovial capsule" OR "Hypertrophic synovial plica" OR "Hypertrophic plica") AND (“Knee” OR “Anterior knee pain” OR “Knee joint” OR “Patellofemoral joint” OR “Tibiofemoral joint” OR “Joint, Knee” OR “Joints, Knee” OR “Joint capsule” OR “Articular capsule" OR “Joint capsule”)

We included all interventional and observational studies (clinical trials, quasi-experiments, case reports, case series) and excluded conference papers, editorials, letters, commentary, short communications, notes, and non-English papers. We followed the principles of the Preferred Reporting Items for Systematic Reviews and Meta-analyses (PRISMA) [19].

**Fig. 6:**
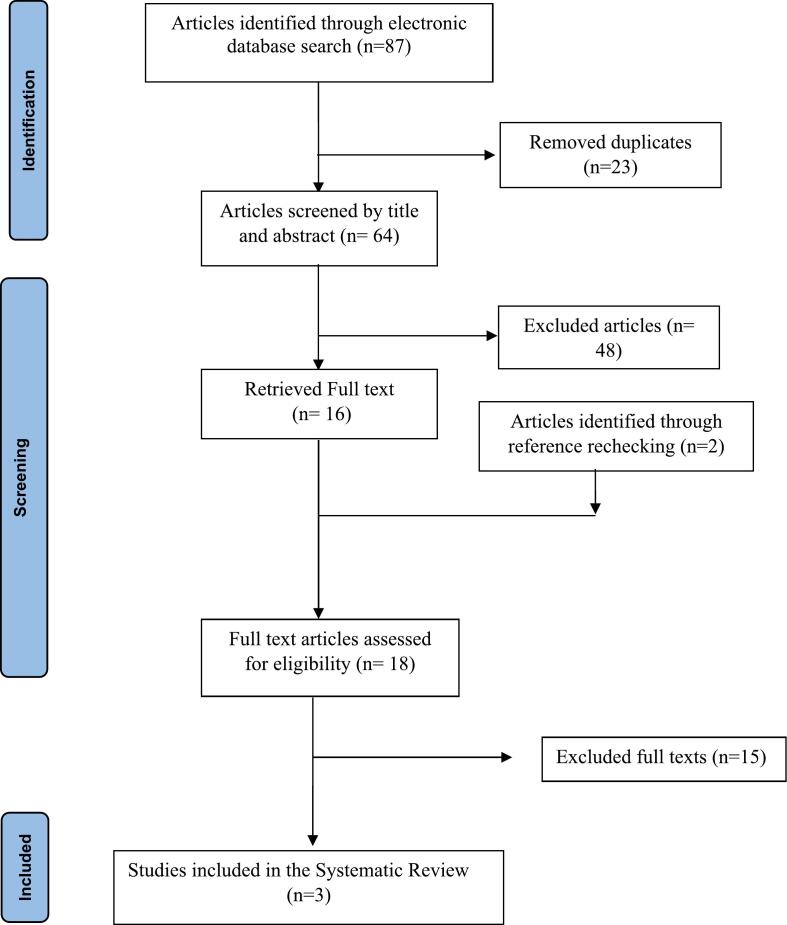
PRISMA flow diagram for identification and selection of the studies.

A total of 87 articles were identified initially. After duplicate removal, two independent researchers screened 64 articles by title and abstract and selected 16 relevant titles for full-text assessment. Two more papers were added after checking the reference lists of the included studies. Of 18 articles that underwent full-text assessment, three titles were finally included in the review (Fig. 6). Other studies were excluded due to their ineligibility. For example, they had another problem besides plica or because they had different symptoms. Table 1 provides a summary of the included articles.

**Table 1:** Summary of included articles.

Study	Period between symptoms to treatment	Patient and age	Symptoms	Unilateral or bilateral	Surgical technique for treatment
Kosaka, (2019, Japan)	5.6 months	10 patients, Average age was 19.1 years old	• Anterolateral knee pain • Limited range of motion due to pain • Catching	Unilateral	•Lateral synovial plica was resected using a shaver and punch during arthroscopy
Kurosaka (1992, Japan)	Not mentioned	A 21-year-old volleyball player	• Pain in both knees during high school sports, episodic and progressive, with a painful click • No history of trauma or other inciting factor	Bilateral	•Arthroscopic resection of abnormal plica tissue with power instruments
Kanazawa (2017, Japan)	6 months	A 15-year-old competitive soccer player	• Anterolateral pain and catching sensation in both knees • Tenderness along the joint line of both patellofemoral joint (PFJ)	Bilateral	• Hypertrophied tissues were debrided with a combination of a punch and an electrocautery abrasion device • The plica was excised using arthroscopic instruments
Our study	2 years	12-years old-male	• Anterior knee pain • Regional pain in the supra-lateral site of the patella in a 90° flexion position • Point tenderness in the condyle in the knee 90° flexion position • No relationship between pain and activities • Mild click during flexion	Unilateral	•Arthroscopic resection

Kosaka et al., in a case series, reported lateral plica in ten athlete patients with a mean age of 19.1 years old. Their symptoms were anterolateral knee pain and decreased knee ROM due to pain. The mean time between symptom onset and surgery was 5.6 months. All of their cases were successfully treated by arthroscopic lateral plica resection [20].

Kurosaka et al. also reported bilateral symptomatic lateral synovial plica in a 21-year-old volleyball player. There was no history of significant trauma. The patient suffered from episodic pain and snapping. Their case was treated successfully with arthroscopic resection [11].

Kanazawa et al., in a case report, represented a 15-year-old soccer player who suffered from bilateral anterolateral knee for six months. Bilateral arthroscopic resection was conducted for this patient successfully [12].

## 4 Conclusion

We recommend that physicians should consider the possibility of synovial hypertrophic plicae, especially in younger patients with histories of direct traumatic events.

## Ethical approval

The study is exempt from ethical approval in institution. Because this study is a case report based on anonymized patient data collection, with the treatment which is according to the guideline. The study adheres to the principles outlined in the Declaration of Helsinki, and ethical principles were followed in the conduct and reporting of this case report. The need for approval was waived by the institutional policy on exempt research. The exemption was confirmed by the ethics committee, and it is relevant and applicable to the current study submitted.

## Funding

This research did not receive any specific grant from funding agencies in the public, commercial, or not-for-profit sectors.

## Author contribution

Ramin Shayan-moghadam and Fardis Vosoughi contributed to the study conception and designing study. Data collection and editing manuscript were performed by Alireza Mirzamohamadi. Erfan Babaei nejad contributed to data collection and designing search strategy. Extracting data and writing the first draft of manuscript was done by Arash Sherafatvaziri. Eligible articles and extracted data were rechecked by Alireza Mirzamohamadi and Erfan Babaei nejad. all authors commented on previous versions of the manuscript. All authors read and approved the final manuscript.

## Guarantor

Ramin Shayan-Moghadam

## Consent

Written informed consent was obtained from the patient and his parents for publication and any accompanying images. A copy of the written consent is available for review by the Editor-in-Chief of this journal on request.

## Conflict of interest statement

The authors declare no conflict of interest and no Funding for the current study.
